# Methylation of HPV and a tumor suppressor gene reveals anal cancer and precursor lesions

**DOI:** 10.18632/oncotarget.17984

**Published:** 2017-05-18

**Authors:** Attila T. Lorincz, Mayura Nathan, Caroline Reuter, Rhian Warman, Mohamed A. Thaha, Michael Sheaff, Natasa Vasiljevic, Amar Ahmad, Jack Cuzick, Peter Sasieni

**Affiliations:** ^1^ Centre for Cancer Prevention, Wolfson Institute of Preventive Medicine, Queen Mary University of London, EC1M 6BQ, UK; ^2^ Homerton Anal Neoplasia Service, Homerton University Hospital NHS Foundation Trust, London E9 6SR, UK; ^3^ Cellular Pathology, Barts Health NHS Trust, London E1 2ES, UK; ^4^ National Bowel Research Centre, Blizard Institute, Queen Mary University of London, London E1 2AT, UK; ^5^ Barts Anal Neoplasia Centre, Department of Colorectal Surgery, Surgery and Cancer CAG, The Royal London Hospital, Barts Health NHS Trust, Whitechapel, London E1 1BB, UK

**Keywords:** anal cancer, intraepithelial neoplasia, DNA methylation, high-risk human papillomavirus, HPV genotyping

## Abstract

We studied DNA methylation patterns of human papillomavirus (HPV) and tumor suppressor gene *EPB41L3* in 148 anal and perianal biopsies to determine whether high levels of methylation would be associated with anal intraepithelial neoplasia (AIN). The most prevalent HPV type was HPV16, detected in 54% of the 30 benign biopsies, 33% of the 43 low-grade AIN (lgAIN), 82% of the 59 high grade AIN (hgAIN) and 4 of the 5 anal cancers. A methylation score was developed (0.561*HPV16me+0.439*EPB41L3) which had increasing values with severity of disease: the mean was 8.1% in benign, 13.2% in lgAIN, 22.3% in hgAIN and 49.3% in cancers (*p* < 0.0001). The methylation score as a triage classifier at a cut-off of 8.8 gave a sensitivity of 90.6% (95% CI: 82.8, 96.9), specificity of 50.7% (95% CI: 39.7, 61.6) and area under the curve of 0.82 (95% CI: 0.75–0.89) for separating hgAIN and cancer from benign and lgAIN biopsies. We conclude that methylation of HPV16 and *EPB41L3* show highly significant association with increasing severity of AIN and cancer and may be useful as biomarkers in anal disease.

## INTRODUCTION

Human papillomavirus (HPV) infects a majority of people worldwide. Infection can occur at any age and can either be transient (usually resolving within a few years) or could be persistent and last for many decades [1]. High risk HPV (hrHPV) infection with types 16, 18, 31, 33, 35, 39, 45, 51, 52, 56, 58, 59, and 68 in epithelial basal cells, especially in certain sites such as the uterine cervix, vulva, vagina, anus, and tonsils is an important risk factor for the development of squamous cell cancers and adenocarcinomas [1, 2]. Natural infections can produce immunity to identical and related HPV types while vaccination with virus-like-particles elicits a strong humoral immune response that is an effective prophylaxis [3]. Persistence of hrHPV is a known strong risk factor for cervical cancer [4] and occurs in immunocompetent individuals but is more common in immunosuppressed patients, such as those infected by HIV [5]. The molecular mechanisms of transient versus persistent hrHPV infections have been only partially elucidated, but may involve differences in integration of the HPV genome into host DNA and DNA methylation [6, 7]. HPV DNA testing can identify almost all prevalent high-grade cervical intraepithelial neoplasia (CIN2 and CIN3) and cervical cancers in exfoliated cervical cells [8]. The test also has a good ability to predict incident disease several years in advance of clinical manifestation [9]. Recent widespread recognition that hrHPV testing is much more sensitive than cytology has driven implementation of primary hrHPV screening for cervical disease in many countries [8].

Anal cancer has been growing in incidence in the past few decades, especially in women and also in men who have sex with men (MSM). Furthermore, anal cancer incidence is higher in HIV-positive MSM with approximately 100 cases compared to 25 cases per 100,000 in HIV-negative MSM [10, 11] and only 1.5 per 100,000 in men in general in the UK [12]. Most anal cancers have been associated with HPV16, while other hrHPV types such as HPV18, HPV31 and HPV33 seem to play a much smaller role in anal cancer than in cervical cancer [13]. High-grade AIN (alternatively called anal HSIL) is also associated with hrHPV, especially HPV16, and multiple HPV types are often reported in HIV-positive men [14].

Anal cytology sampling is problematic because anal folds may hide lesions and this has resulted in recommendations for more frequent sampling to compensate for poor sensitivity [15]. Normal, borderline or mildly dyskaryotic (also called ASCUS or LSIL) cytology is common in patients with hgAIN [16]. High-resolution anoscopy (HRA) is often used as the primary screening tool for high-risk populations in settings where resources can support such an intensive approach [17]. However, besides the high cost, using HRA to detect hgAIN has additional limitations such as a subjective result, availability of a trained HRA specialist and discomfort caused to the patient. Consequently, for decades, other methods of triage to biopsy and treatment have been actively sought to lessen the burden on the HRA clinics [18].

DNA methylation testing of HPV and human genes has been validated as an accurate method for detection of CIN2 and CIN3 [19–22]. Levels of methylation increase over time in women with persistent HPV16 infection and are maximal in patients with cancer [23, 24]. We investigated if a similar methylation test might be usefully applied to people with anal disease based on our *a priori* hypothesis that high levels of methylation at genomic positions associated with hgCIN would also be associated with hgAIN. Here, we focus on the methylation of host gene *EPB41L3* and the high risk viral types: HPV16, HPV18, HPV31 and HPV33. *EPB41L3* (Erythrocyte Membrane Protein Band 4.1 like 3) is a tumor suppressor gene that inhibits cell proliferation, promotes apoptosis and has been found to be highly methylated in many cancers such as lung, cervix, ovarian and breast [25–28].

## RESULTS

There were 30 biopsies with <AIN, 43 lgAIN, 59 hgAIN and 5 cancers among the anal samples and 11 biopsies of high-grade perianal lesions (Table 1).

**Table 1:** HPV typing data of anal and perianal samples by lesion type

		HPV16	Other hrHPV types	lrHPV types or negative	Total
		*n* (%)	*n* (%)	*n* (%)	*n*
Anal	<AIN	16 (53.3)	4 (13.3)	10 (33.3)	30
	lgAIN	14 (32.6)	15 (34.9)	14 (32.6)	43
	hgAIN	49 (83.0)	5 (8.5)	5 (8.5)	59
	Cancer	4 (80.0)	1 (20.0)	0 (0)	5
	Total	83 (60.6)	25 (18.2)	29 (21.2)	137
Perianal	hgAIN	10 (90.9)	1 (9.1)	0 (0)	11

Other hrHPV: high-risk and possibly high risk HPV other than type 16 (this includes HPV18, 31, 33, 35, 39, 45, 51, 52, 56, 58, 59, 66, 68); lrHPV: low-risk HPV (HPV6 and 11); AIN: anal intraepithelial neoplasia; lg: low-grade; hg: high-grade.

### HPV genotyping

About a third (47/148) of samples were infected with multiple HPV types. 33% of anal biopsies with either <AIN, or lgAIN histopathology were not infected by hrHPV types while 53% and 33%, respectively, were HPV16 positive (Table 1). hgAIN anal and perianal biopsies were predominantly infected by HPV16 (83% and 91% respectively). A small proportion (9%) of anal and perianal hgAIN were infected with hrHPV types other than HPV16, i.e. 5 out of 59 of anal and 1 out of 11 of perianal biopsies. Also of note, all the perianal lesions were hrHPV positive. Only 9% of anal hgAIN biopsies were not infected by hrHPV (5/59). All cancers were hrHPV positive, four with HPV16 and one with HPV33.

### DNA methylation

**Figure 1:**
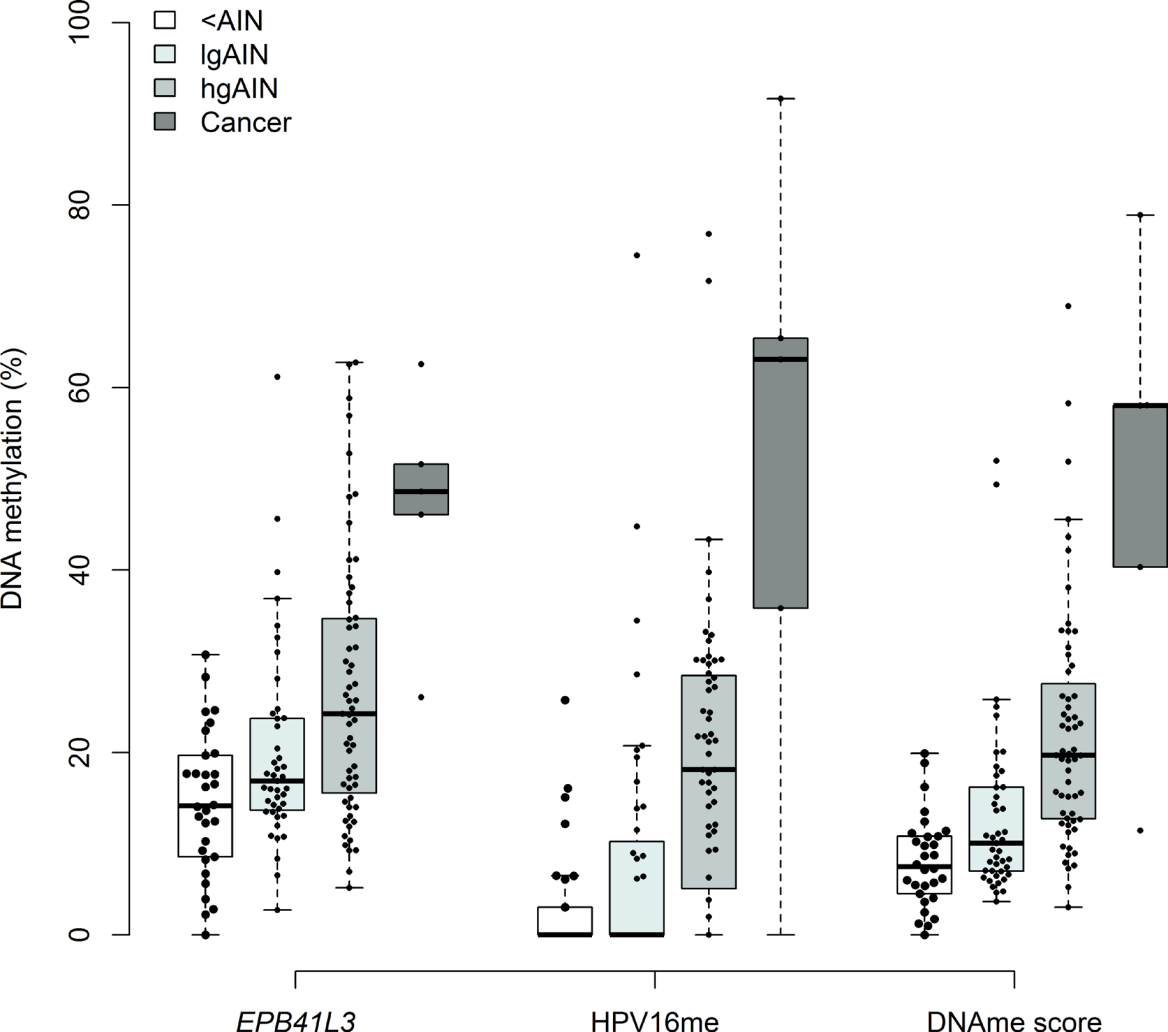
Comparison of DNAme levels of *EPB41L1*, HPV16 and the DNAme score (0.561*HPV16+0.439*EPB41L3) in <AIN, lgAIN, hgAIN and cancer cases Perianal samples are not included in this figure. The top of box represents the upper quartile, bottom the lower quartile and line the median. The upper (lower) whisker extends to the largest (smallest) point that is not more than 1.5× of the inter-quartile range from the upper (lower) quartile. All data points with a methylation value > 0 are shown individually (black circle).

The DNAme levels of *EPB41L3*, HPV16L1 and HPV16L2 were significantly different between the four groups and increased with severity of the lesions (*p* < 0.0001, Cuzick test for trend), but no significant differences in the likelihood ratios were found for methylation of HPV18, HPV31 and HPV33 and these latter markers were dropped from further analysis (Table 2). The univariable models (Table 2) investigating *EPB41L3*, HPV16L1 and L2 regions were all highly significant (*p* < 0.0001). The bivariable logistic regression using *EPB41L3* and HPV16me was highly significant (*p* < 0.0001, Table 2) as was each variable on its own. The linearly combined DNAme score was derived from the bivariable model and calculated as follows: 0.561*HPV16me+0.439*EPB41L3. For all three variables (*EPB41L3*, HPV16me and the DNAme score), there was a highly significant trend of increased methylation with disease progression (Cuzick tests for trend, *p* < 0.0001). Figure 1 shows the methylation of *EPB41L3*, HPV16me and the DNAme score.

**Table 2:** Univariable and bivariable logistic models of DNA methylation, age and gender

Logistic models					
Univariable				Bivariable	
Markers	*N*	IqrOR^a^ (95% CI)	Model LR χ^2^ (*p*)	OR^a^ (95% CI)	Model LR χ^2^ (*p*)^b^
DNAme score^c^	137	5.454 (2.664, 11.154)	39.036 (4.16e-10)		
HPV16me	137	4.866 (2.454, 9.647)	31.573 (1.92e-08)	1.066 (1.030, 1.104)	31.573 (1.92e-08)
HPV16me L2	137	3.689 (1.914, 7.112)	25.466 (4.5e-07)		
*EPB41L3*	137	2.836 (1.721, 4.676)	22.292 (2.34e-06)	1.052 (1.012, 1.092)	7.463 (0.0063)
HPV16me L1	137	3.306 (1.708, 6.401)	14.286 (1.57e-04)		
Age	136	1.769 (1.069, 2.927)	5.221 (0.0223)		
HPV31me	137	0.101 (0.008, 1.348)	3.832 (0.0503)		
HPV33me	137	9.562 (0.478, 191.242)	1.531 (0.2160)		
Gender	136	0.576 (0.236, 1.407)	1.487 (0.2227)		
HPV18me	137	1.013 (0.982, 1.045)	0.692 (0.4054)		

^a^IqrOR and OR for a 1% change in DNA methylation, ^b^variables added sequentially (first to last), ^c^The score was developed as a linear predictor of the bivariable logistic regression, me: methylation.

DNA methylation is presented for the newly developed score (DNAme score), EPB41L3, HPV16, HPV18, HPV31 and HPV33. For HPV16, the methylation levels of two regions, L1 (HPV16me L1) and L2 (HPV16me L2), and the average of both regions (HPV16me), are presented. The DNAme score is calculated using (0.561*HPV16me+0.439*EPB41L3) to predict high-grade anal neoplasia. Odds ratios (OR), inter-quartile odds ratios (IqrOR), likelihood ratios (LR), χ^2^ and p-values of the models are given.

**Figure 2:**
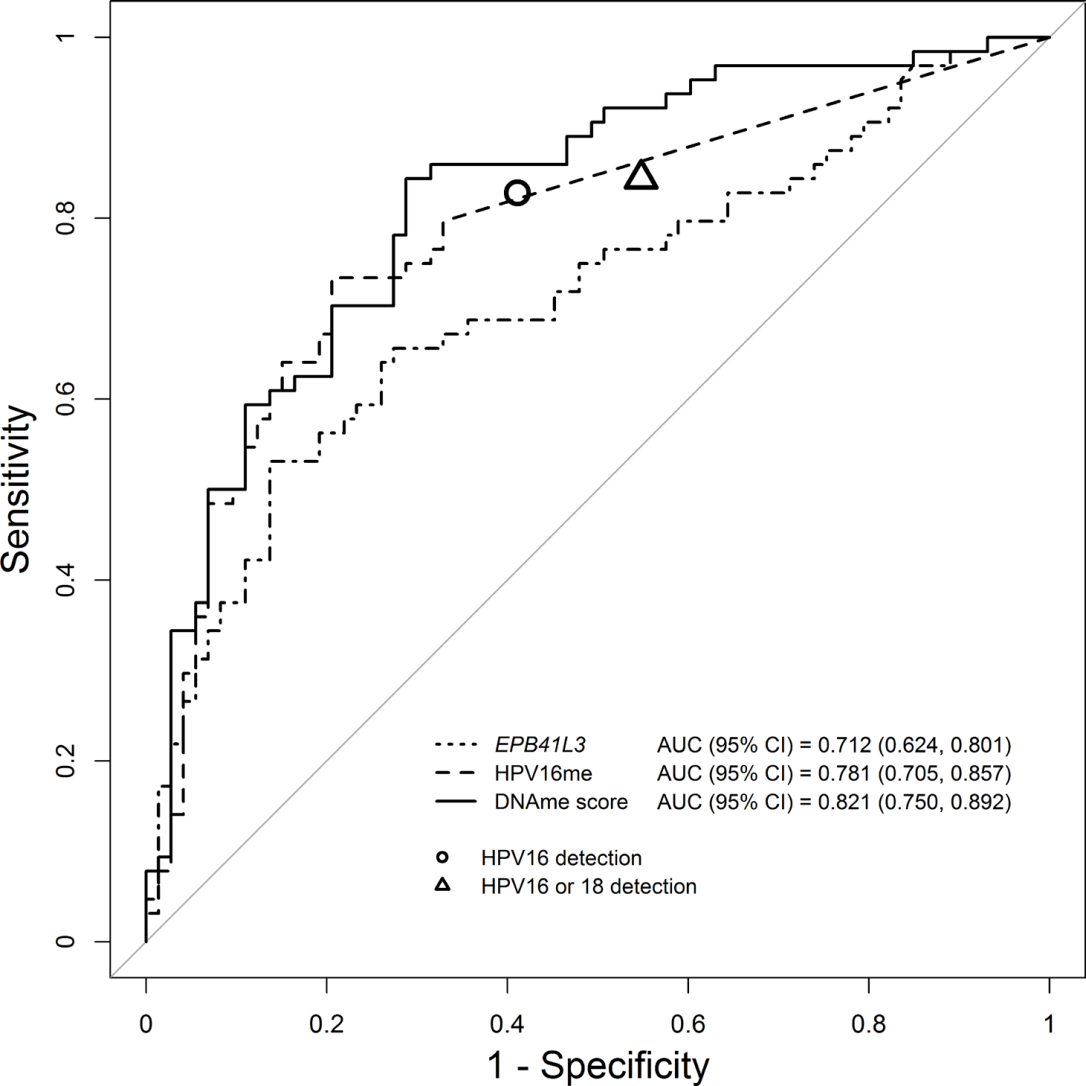
Receiver operator characteristic and associated area under the curve (AUC) of DNAme of *EPB41L3*, HPV16 and the DNAme score The DNAme score (solid line) performed significantly better than *EPB41L3* (dotted line) or HPV16 methylation (dashed line) on their own. For comparison the circle represents the performance of HPV16 genotyping while the triangle represents the performance of combined genotyping for HPV16 or HPV18.

The ROC curves comparing the methylation levels in the <AIN and lgAIN samples to the hgAIN and cancer cases (Figure 2) had an AUC of 0.712 (95% CI: 0.624, 0.801, *p* < 0.0001) for *EPB41L3*, 0.781 (95% CI: 0.705, 0.857, *p* < 0.0001) for HPV16me and 0.821 (95% CI: 0.750, 0.892, *p* < 0.0001) for the DNAme score. Figure 2 also shows the relative sensitivities and specificities of genotyping for HPV16 or genotyping for HPV16 and HPV18 combined. Supplementary Figure 1 shows ROC curves comparing missing DNAme values imputed by MICE versus single imputation. The chart indicates that there was no statistically significant difference in our interpretations of the data using either the multiple or single imputed data.

Sensitivity and specificity of the DNAme score to detect hgAIN and cancers and the proportion of positive samples identified by the DNAme score is shown in Table 3. At the 7.5 cut-off the DNAme score correctly identified all the cancers and 95% of the hgAIN, while correctly classifying 33% of the lgAIN and 50% of the <AIN biopsies. In comparison, sensitivity of HPV16 genotyping to detect hgAIN and cancer was 83% (95% CI: 73–92) and specificity was 59% (95% CI: 48–70).

**Table 3:** Sensitivity and specificity at specified cut-offs to detect hgAIN and cancers and proportion of positive samples identified by the DNAme score

					Proportion of samples identified by the DNAme score			
Cut-off	% Sensitivity (95% CI)	% Specificity (95% CI)	χ^2^	*p*-value	<AIN	lgAIN	hgAIN	Cancer
7.5	95.3 (90.6–100)	39.7 (28.8–50.7)	25.426	4.60e-07	0.50	0.67	0.95	1.00
8.8	90.6 (82.8–96.9)	50.7 (39.7–61.6)	27.872	1.30e-07	0.40	0.56	0.90	1.00
10.3	85.9 (76.6–93.8)	58.9 (47.9–69.9)	42.117	8.60e-11	0.20	0.40	0.85	1.00

The 95% confidence intervals (95% CI) were computed using 2000 stratified bootstrap replicates. AIN: anal intraepithelial neoplasia; lg: low-grade; hg: high-grade.

Supplementary Figure 2 shows a scatter plot of methylation of *EPB41L3* against HPV16me with the relationship having a weak Spearman correlation. We also investigated whether the DNAme score would be able to correctly identify the 11 high-grade perianal samples (Supplementary Figure 3) using cut-offs obtained with anal samples. Ten of the cases were correctly identified at the cut-off 7.5 and nine at the cut-off 8.8. Finally, we stratified the methylation data by HPV16 positivity and found that most of the predictive methylation information was in the HPV16 infected patients, which demonstrated that DNA methylation provided triage information in addition to the information given by HPV16 genotyping. In contrast little if any diagnostic contribution was seen for methylation in the HPV16 negative group (Supplementary Figure 4).

## DISCUSSION

The *a priori* hypothesis that high levels of methylation at genomic positions shown to be associated with hgCIN in our earlier research [19, 29] would also be associated with hgAIN has been confirmed, thus opening the way to methylation diagnostics of anal disease. Anal cytological abnormalities are poor predictors of hgAIN amongst HIV-positive patients [30]. Moreover, there is not a good correlation between cytology grades and histology grades. There are also substantial differences between pathologists in interpreting anal histology. In our study, we used a single pathologist (MS) who had extensive experience in anal pathology for all histology and the diagnoses were backed up by p16 staining when indicated [31, 32]. Goldstone et al. showed that 20% of patients with normal anal cytology and more than 30% with borderline cytology had hgAIN [33]. HPV DNA testing as a primary anal screen has the advantage of greater sensitivity than cytology and has an advantage of lower costs than screening with high-resolution anoscopy. Another advantage of HPV screening is that anoscopy is a complex procedure with a need for extensive training of practitioners. However, the fundamental problem with HPV DNA testing that has precluded its widespread use in identifying anal precancer is poor specificity. For example, Salit et al. [17] showed that 88% of their HIV+ patients were HPV DNA positive for carcinogenic types. The specificity of HPV testing can be partially rectified by focusing on HPV16, which is one of the most common types found in anal cancer [34–37]. However, since HPV16 is not present in all anal cancers there is a good chance that high-risk progressive lesions will not be detected. In our study, we found that most AIN were positive for hrHPV DNA and the majority of hgAIN were positive for HPV16, with relatively few positive for other hrHPV types. However, only 4 of the 5 anal cancers were positive for HPV16, the other being positive for HPV33, which shows the limitation of relying on HPV16 genotyping triage.

DNA methylation is a potential option that may offer greater improvements in triage specificity while retaining good sensitivity and importantly detect all the cancers. In our study, all of the anal cancers were positive (i.e. above the cut-off) for DNA methylation, similar to what has been generally observed for cervical cancer. The DNAme score we developed is a multi-biomarker panel composed of three CpG sites within the *EPB41L3* gene and the late regions (L1 and L2) of HPV16. The AUC of the methylation score for separating <AIN and lgAIN from hgAIN and cancer was 0.82 (95% confidence interval: 0.75, 0.89, *p* < 0.0001, Table 3). Studies have shown that cervical cancers have higher levels of methylation than CIN3 [20]. All the anal cancers in our study were highly methylated and quite well separated from the hgAIN. This suggests the possibility that DNA methylation may be used to indicate AIN destined to progress to anal cancer from lesions that will regress or remain indolent [38–40].

It is notable that the triage information for hgAIN and cancer provided by the methylation score was significantly higher only in HPV16 positive people (Supplementary Figure 4); however, the cancer negative for HPV16 was strongly methylated (25%) for *EPB41L3*. These data demonstrate that DNA methylation provides significant diagnostic information for detecting hgAIN in addition to that provided by HPV16 genotyping and suggests that a methylation test may be used to detect essentially all the cancers. The methylation score in HPV16 negative people did not show a significant discriminating effect. However, we cannot be sure of the lack of value of methylation information in the HPV16 negative samples because the analysis was underpowered for this endpoint. These results indicate the need for larger studies and a search for more genes that could provide additional triage information in people infected with hrHPV types other than HPV16.

A limitation of the study is that we used FFPE biopsies. Our results need to be replicated in an adequately powered study of exfoliated anal cells because in routine practice hrHPV positive patients would have methylation tests performed on exfoliated cells collected by a swab or similar device. This would allow efficient triage to HRA, thus reducing costs, anxiety and possible over-treatment of low risk people. Our study had incomplete information on HIV status and the small size of the HIV-negative subset likely produced some ascertainment bias. Another limitation of our study is the use of patients (mostly MSM) recruited from two sites specializing in anal HPV-related disease in London. It remains to be seen if our results can be duplicated in other settings.

We conclude that high levels of DNA methylation are associated with hgAIN and anal cancer. This finding should be further explored to better understand the biological mechanisms and the value of DNA methylation testing as a molecular triage of hrHPV positive individuals for high-resolution anoscopy screening.

## MATERIALS AND METHODS

### Patients

A set of anal and perianal biopsy specimens were obtained from 148 patients (116 men, 31 women, and 1 person of unrecorded gender) of whom 94 were HIV positive, 40 were HIV negative and 14 had not been tested for their HIV status (Supplementary Table 1). The formalin-fixed paraffin-embedded (FFPE) biopsies were retrieved from the archives of the Homerton University Hospital and St Bartholomew's and the Royal London Hospital in London, UK, which are tertiary referral units where people with suspected hgAIN are referred for further management. Institutional approval (R&D number: GU1310) for the study was obtained prior to commencing any research work on the specimens.

In patients undergoing HRA, biopsies were obtained from areas of clinical interest that exhibited acetowhite changes (regardless of vascular changes) on 5% acetic acid application. Most of the biopsies showed morphological changes varying from slight to severe which distinguished them from the surrounding non-acetowhite normal epithelium. The main clinical endpoint for all comparisons was the histology result. The biopsies were graded histopathologically using the AIN terminology [41] which is in general use in the UK, acknowledging that recent recommendations for terminology in the US distinguish between low grade squamous intraepithelial lesion (LSIL equivalent to AIN1) and HSIL (equivalent to AIN2 and AIN3). The histopathological diagnoses were based on expert review by one of our team (MS) who has worked on anal neoplasia for more than 10 years. Diagnostic adjudication of difficult AIN cases was assisted by p16 staining of tissue sections [42] using the following rule: if the p16 result was positive (diffuse staining), the higher diagnosis (hgAIN) was assigned and if p16 was negative (focal, sporadic and negative staining), the lower diagnosis (lgAIN) was assigned. However, some of the biopsies showed only presence of HPV, and other biopsies showed slight changes that did not fulfil the criteria for lgAIN or hgAIN. These evidently non-normal biopsies are assumed as benign and were graded as <AIN.

### HPV genotyping

We used H&E sections annotated by MS as a guide to dissect areas of interest on the corresponding four unstained 5 μm sections, as previously described [43]. If more than one lesion was present on a single section, we dissected the lesion with the highest grade. The dissected areas from the four sections were placed in the same tube. Dissected areas were deparaffinized using 80 μL of hexadecane followed by a 5-minute incubation at 56°C. One hundred microliters of universal extraction buffer containing 50 mM Tris-HCl pH 8.0, 1 mM EDTA and 0.05% SDS [44] was added to tissues along with 10 μl of Proteinase K (QIAGEN) and incubated overnight at 56°C followed by a one-hour incubation at 90°C. The lower phase was then transferred to a new tube and stored at −20°C before PCR.

The samples were tested using the PapType High Risk HPV Detection and Genotyping kit (PapType kit, Genera Biosystems Ltd) according to the manufacturer's instructions. The kit is able to detect 13 high-risk HPV types (HPV16, 18, 31, 33, 35, 39, 45, 51, 52, 56, 58, 59, and 68) a possible high risk type (HPV66) and two low-risk types (HPV6 and HPV11). The PapType test was performed with 10 μL of DNA in a final reaction volume of 20 μL with the addition of 2% Tween 20. The PCR reaction amplifies a variable region of the L1 gene of the HPV genome. A fragment of the human cardiac myosin light chain gene (MLC-1) was co-amplified in the same reaction vessel as a quality and quantity control. For simplicity, we categorized the samples into three HPV genotype groups independently of whether they were singly or multiply infected: those infected by (1) HPV16, (2) by any other high-risk types (including HPV66) and (3) those HPV negative or infected only by a low-risk type (lrHPV). We did not combine HPV18 with HPV16 due to obvious differences in viral disease characteristics as noted in earlier studies [1, 45] and the fact that HPV18 does not seem to play such an important role in anal cancers [46].

### Methylation assays

Bisulfite conversions on 20 μl of DNA extracts were done using EZ DNA Methylation Kit (Zymo Research, CA, USA) following the manufacturer's instructions. DNA methylation (DNAme) was measured by pyrosequencing for human biomarker *EPB41L3* and viral late genomic regions of HPV16 (CpG sites in the L1 region: 6367, 6389 and L2 region: 4238, 4247, 4259, 4268, 4275) and HPV18 (CpG sites in the L2 region: 4257, 4262, 4266, 4269, 4275, 4282), HPV31 (CpG sites in the L1 region: 6352, 6354) and HPV33 (CpG sites in the L2 region: 5557, 5560, 5566 and 5572) as previously described [47]. Amplification of CpG sites were carried out using PyroMark PCR kits (QIAGEN, Germany) with 4 μl (8 μl) of converted DNA for *EPB41L3* (HPV assays) in a 25 μl volume with final concentration of reagents of 1× for Coral Load and PyroMark mix, 0.2 μM of PCR primers. PCR cycling conditions were 15 minutes at 94°C, followed by 45 cycles of 94°C, 54°C, 72°C each for 30 seconds and a final extension at 72°C for 10 minutes. The PCR products were pyrosequenced using a PyroMark^™^Q96 ID (Qiagen) instrument as previously described [48]. All pyrosequencing runs included a negative control and positive controls of known methylation level (0%, 50% and 100%) to allow standardized direct comparisons between different primer sets. For each marker, we calculated the average methylation level by taking the mean of all CpG positions.

### Statistical methods

Missing methylation values for HPV were imputed with the value of zero for any HPV negative sample. Missing methylation values for *EPB41L3* and HPV positive samples were imputed using a median regression with age as a predictor and DNA methylation as an outcome. All statistical analyses were performed on the imputed data set [49]. Out of the 137 anal samples, six had missing methylation values for *EPB41L3*, 22 for HPV16L1, 31 for HPV16L2, 5 for HPV18, 11 for HPV31 and none for HPV33. Of the 11 perianal samples, 4 had missing values for HPV16L1, 1 for HPV16L2 and 3 for HPV18. No value was missing for *EPB41L3*, HPV31 nor HPV33 (Supplementary Table 2).

Statistical analyses for the anal and perianal lesions were not combined. Spearman correlations were calculated between the markers. Since methylation values of HPV16L1 and HPV16L2 regions were correlated (Spearman *r* = 0.570, *p* < 0.0001), a variable called HPV16me was created by taking the geometric mean of DNAme levels of HPV16L1 and HPV16L2.

Univariable and bivariable logistic models were fitted for statistically significant genes with the outcome measures 0 = <AIN and lgAIN, and 1 = hgAIN and cancer. The likelihood ratio (LR) χ^2^ statistic and its corresponding *p*-value, as well as the odds ratios (OR) with 95% confidence intervals were estimated and reported. Performance of the markers were assessed univariably by the LR χ^2^ test and the area under the curve (AUC) with 95% confidence intervals (CI) using the Delong method [50]. Sensitivity and specificity were estimated at selected cut-offs (i.e. cut-off values giving the same sensitivity as HPV16 genotyping as well as at 90% and 95% sensitivity).

A combined DNAme score of *EPB41L3* and HPV16me was computed as a linear predictor of the fitted bivariable logistic regression. Confidence intervals for difference in sensitivities and specificities between the DNAme score at different cut-offs and HPV16 genotyping was computed using 2000 stratified bootstrap replicates as recommended by Carpenter and Bithell [51]. Cuzick tests for trend were applied to *EPB41L3*, HPV16me and the DNAme score to test for significant changes of methylation between the groups (<AIN, lgAIN, hgAIN, and cancer).

Multiple imputations analyses were performed to test whether the single imputation led to any bias [52]. We used the multivariable imputation by chained equations (MICE) procedure (Classification and Regression Trees, CART) [53] with m = 100 multiple imputations [54]. Rubin's rules were used to combine the multiply imputed estimates [55]. Receiver operating characteristics (ROC) of the DNAme score and AUC (95% CI) were estimated for the 100 multiple imputations separately and for their average.

All *p*-values were two-sided with significance set at α < 0.05. Analyses were undertaken using R statistical software version 3.3.1 [56].

## SUPPLEMENTARY MATERIALS FIGURES AND TABLES



## Abbreviations

AIN: anal intraepithelial neoplasia
ASCUS: Atypical Squamous Cells of Undetermined Significance
AUC: area under the curve
CART: Classification and Regression Trees
CI: confidence interval
CIN: cervical intraepithelial neoplasia
CpG: 5′—Cytosine—phosphate—Guanine—3′
DNAme: DNA methylation
EDTA: Ethylenediaminetetraacetic acid
FFPE: formalin-fixed paraffin-embedded
HCl: Hydrochloric Acid
H&E: Hematoxylin and Eosin
hgAIN: high-grade anal intraepithelial neoplasia
hgCIN: high-grade cervical intraepithelial neoplasia
HIV: human immunodeficiency virus
HRA: High-resolution anoscopy
hrHPV: high-risk human papillomavirus
HSIL: high-grade squamous intraepithelial lesion
HPV: human papillomavirus
IQR: interquartile range
lgAIN: low-grade anal intraepithelial neoplasia
LR: likelihood ratio
lrHPV: low-risk human papillomavirus
LSIL: low-grade squamous intraepithelial lesion
MICE: multivariate imputation by chained equations
MLC: human cardiac myosin light chain
MSM: men who have sex with men
OR: odds ratio
PCR: Polymerase Chain Reaction
R&D: research & development
ROC: Receiver operating characteristics
SDS: Sodium Dodecyl Sulfate
Tris: trisaminomethane
UK: United Kingdom.
