# Supplementary material for: Methylation of HPV and a tumor suppressor gene reveals anal cancer and precursor lesions
Source: Oncotarget. 2017 May 18;8(31):50510–20. doi: 10.18632/oncotarget.17984 (PMC5584159; doi:10.18632/oncotarget.17984)
Supplement: Supplementary file 1 [file oncotarget-08-50510-s001.pdf]

Methylation of HPV and a tumor suppressor gene reveals anal cancer and precursor lesions

Supplementary Materials

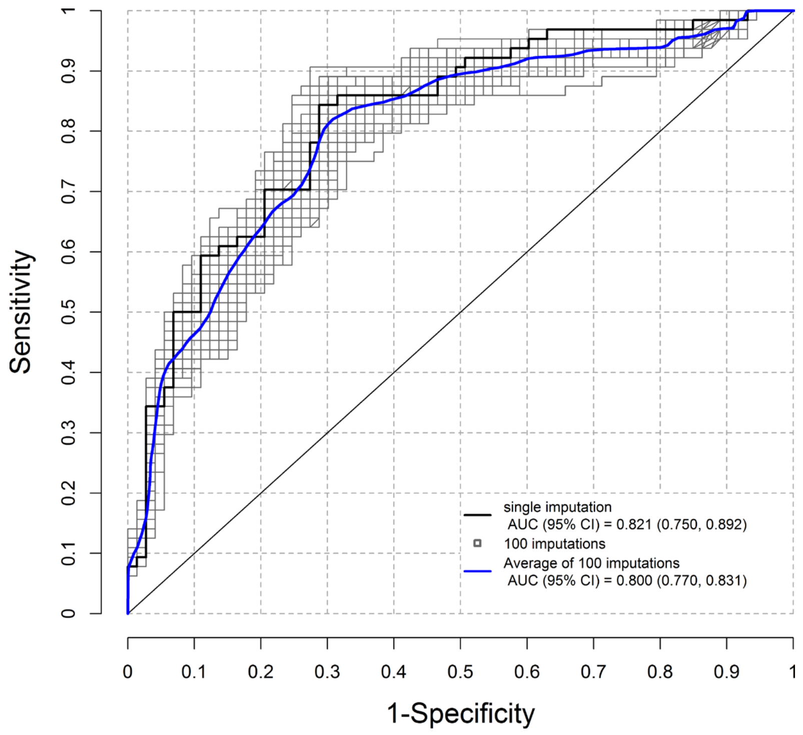

Supplementary Figure 1: Receiver operator characteristic and area under the curve (AUC, 95% CI) of the DNAme score calculated from the single-imputed data set (black), 100 imputed data set (grey) and the average of the 100 imputations (blue).

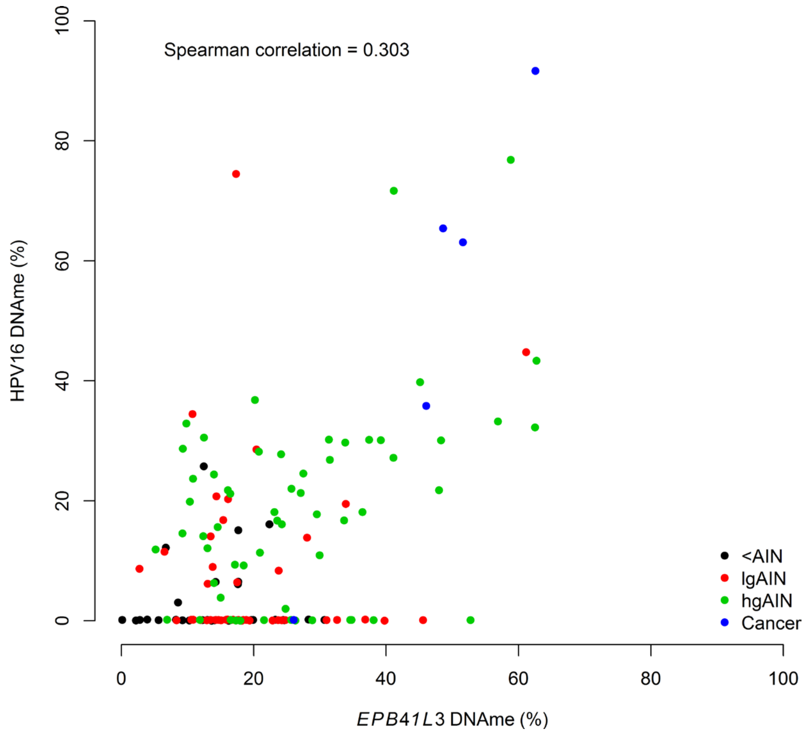

Supplementary Figure 2: Scatterplot of HPV16 versus *EPB41L3* DNA methylation stratified by histopathological diagnosis.

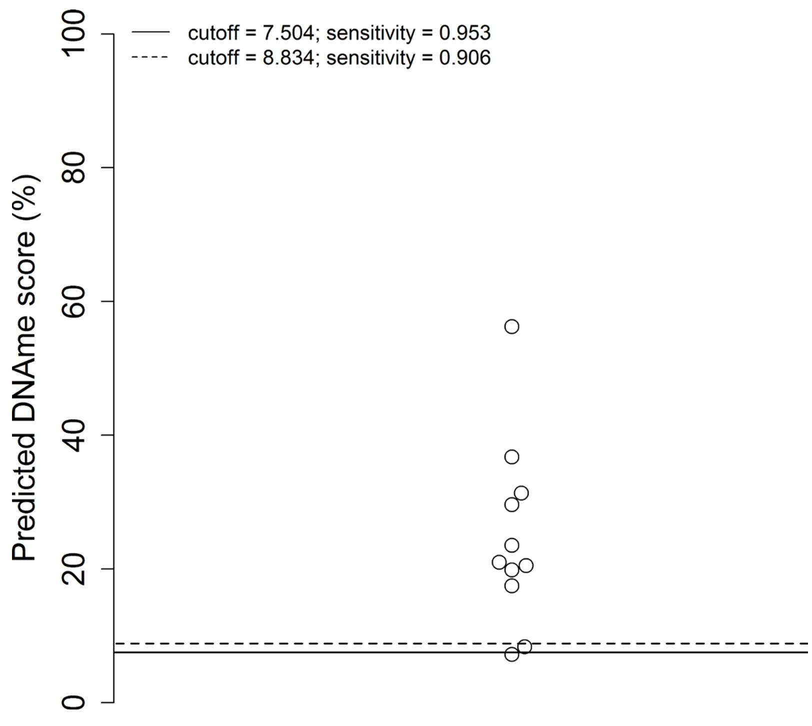

Supplementary Figure 3: Dot plot of the DNAme score (0.561*HPV16+0.439EPB41L3*) calculated for the 11 high-grade perianal samples.**

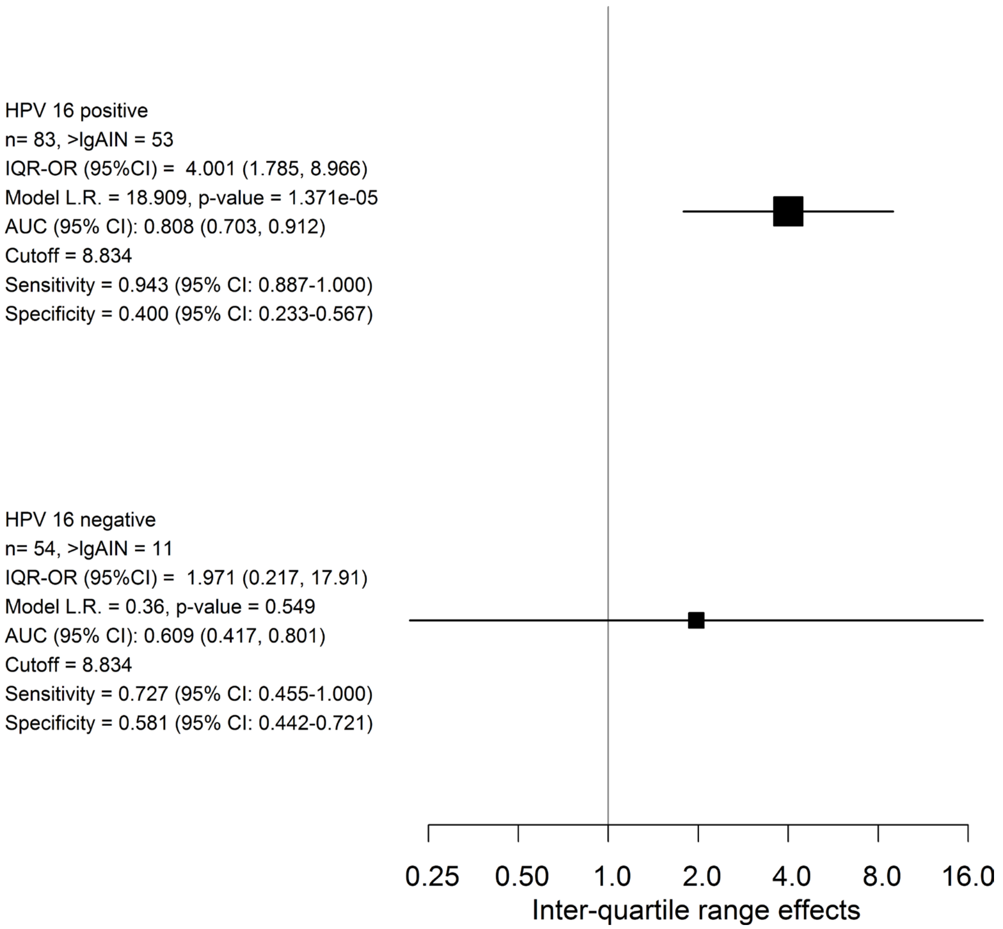

Supplementary Figure 4: Forest plot of the DNAme score stratified by HPV16 positivity for detecting hgAIN and cancer.

Supplementary Table 1: Demographic variables of samples used in the study

Region	Grade	Gender	HIV-negative	HIV-positive	HIV-not tested	hrHPV-negative	hrHPV-positive	Age Median [IQR]
137 Anal biopsies	<AIN = 30	M = 22	9 (41)	8 (36)	5 (23)	8 (36)	14 (64)	41.0 [12.0]
		F = 7	5 (72)	0 (0)	2 (28)	1 (14)	6 (86)	
		U = 1	0 (0)	0 (0)	1 (100)	1 (100)	0 (0)	
	lgAIN = 43	M = 40	8 (20)	32 (80)	0 (0)	14 (35)	26 (65)	43.0 [11.5]
		F = 3	3 (100)	0 (0)	0 (0)	0 (0)	3 (100)	
	hgAIN = 59	M = 46	5 (11)	40 (87)	1 (2)	4 (9)	42 (91)	48.0 [19.5]
		F = 13	7 (54)	2 (15)	4 (31)	1 (8)	12 (92)	
	Cancer = 5	M = 4	1 (25)	3 (75)	0 (0)	0 (0)	4 (100)	59.0 [6.0]
		F = 1	1 (100)	0 (0)	0 (0)	0 (0)	1 (100)	
11 Perianal biopsies	hgAIN = 11	M = 4	0 (0)	4 (100)	0 (0)	0 (0)	4 (100)	46.0 [13.5]
		F = 7	1 (14)	5 (72)	1 (14)	0 (0)	7 (100)	

The table shows the number (percentage) of samples that were HIV negative, positive or not tested and hrHPV negative and positive. AIN: anal intraepithelial neoplasia, lg: low-grade, hg: high-grade, F: female, M: male, U: unrecorded gender, HIV: human immunodeficiency virus, hrHPV: high-risk HPV, HPV: human papillomavirus, IQR: interquartile range.

Supplementary Table 2: Number (percentage) of missing values for the six investigated markers on the 148 biopsies

Marker	Anal, *n* = 137	Perianal, *n* = 11
EPB41L3	6 (4)	0 (0)
HPV16me L1	22 (16)	4 (36)
HPV16me L2	31 (23)	1 (9)
HPV18me	5 (4)	3 (27)
HPV31me	11 (8)	0 (0)
HPV33me	0 (0)	0 (0)

HPV16, HPV18, HPV31 or HPV33 DNA was detected in the samples but the methylation assays failed.
